# Reciprocity? International Preceptors’ Perceptions of Global Health Elective Learners at African Sites

**DOI:** 10.5334/aogh.2342

**Published:** 2019-03-15

**Authors:** Elizabeth M. Keating, Heather Haq, Chris A. Rees, Padma Swamy, Teri L. Turner, Stephanie Marton, Jill Sanders, Edith Q. Mohapi, Peter N. Kazembe, Gordon E. Schutze

**Affiliations:** 1 University of Utah, Department of Pediatric Emergency Medicine, Salt Lake City, UT, US; 2 Baylor College of Medicine, Department of Pediatrics, Houston, TX, US; 3 Boston Children’s Hospital, Harvard Medical School, Division of Emergency Medicine, Boston, MA, US; 4 Baylor College of Medicine Children’s Foundation Lesotho, Maseru, LS; 5 Baylor College of Medicine Children’s Foundation Malawi, Lilongwe, MW

## Abstract

**Background:**

Short-term global health electives (STGHEs) have become increasingly common, with evidence showing educational and clinical benefits for short-term learners (STLs). Despite increased recognition that STGHEs should be mutually beneficial for host sites and STLs, evidence demonstrating the impact on international host preceptors is lacking.

**Objectives:**

To understand international host preceptors’ perceptions regarding benefits and burdens of hosting STLs.

**Methods:**

Focus group discussions with a convenience sample of 10 of 18 eligible preceptors were conducted at pediatric STGHE sites in Malawi and Lesotho. Qualitative content analysis was performed to identify themes using a deductive-inductive approach.

**Findings:**

Common themes regarding benefits to preceptors included increased knowledge and resources for learning from STLs, broadened differential diagnoses, and the satisfaction of teaching. Regarding burdens, preceptors perceived that supervising STLs decreases efficiency. Preceptors identified the burden of having to intervene in instances that could lead to patient harm. Some preceptors perceived that STLs under-valued preceptors’ clinical decision-making in resource-limited contexts.

**Conclusions:**

Our findings emphasize the need for institutions to identify mutuality of benefits between STLs and host sites when developing STGHEs. Host preceptors identified robust pre-departure training for STLs, lengthened duration of STGHEs, and formal preceptor orientation as ways to enhance mutuality of benefits.

## Introduction

Short-term global health electives (STGHEs) have become increasingly common among both medical students and residents and are offered by many institutions in high-income countries [1]. Short-term learners (STLs) are medical trainees (medical students, residents, or fellows) from high-income countries that spend up to six weeks completing STGHEs at host sites around the world. Studies have shown that STLs gain increased cross-cultural communication, enhanced knowledge of tropical diseases, and decreased reliance on laboratory testing and imaging during STGHEs [2,3,4,5,6,7,8].

There is increasing recognition in the global health literature that STGHEs should be mutually beneficial to both host sites and STLs [9]. However, evidence showing benefits to the international host preceptors is lacking. There have been various proposed methods to improve benefits to host sites during STGHEs, such as the creation of programs that allow for bidirectional exchange of learners and asking STLs to bring equipment to the sites, but these ideas have originated from sending institutions [9,10,11,12]. Perspectives from international host sites on how to increase benefits from hosting STLs are lacking.

Due to the lack of data stemming from international host sites, the increasing popularity of STGHEs, and the importance of mutual benefits, this study sought to gain an understanding of preceptors’ perceptions regarding benefits and burdens of supervising STLs, as well as to elucidate ways to increase benefits to host sites.

## Methods

This was a qualitative study using focus group discussions of a convenience sample of host country preceptors that drew upon constructivist grounded theory to identify the perceived impact on host preceptors and their clinical sites of having STLs. The authors selected focus group discussions in order to stimulate open discussion between study participants. The study was conducted at two of the Baylor International Pediatric AIDS Initiative Clinical Centers of Excellence in Malawi and Lesotho. These clinics, which provide outpatient care for children and families with HIV, tuberculosis, malnutrition, malaria, and other conditions, hosted a median of 38 (range 20–47) STLs per year from 2007–2014 from medical schools and residency programs in the United States and Canada [13].

To identify potential study participants, the clinic directors, who oversee clinic operations, provided the study team with a list of all clinical preceptors who had supervised STLs at the respective clinics for at least six months. As the study aimed to capture the opinion of host-country preceptors, all selected participants completed their medical training in low- or middle-income countries. Preceptors who had less than six months of experience working with STLs were excluded. A request for participation was both emailed and verbally communicated to eligible participants, representing convenience sampling. Of 18 eligible preceptors, 10 participated in 2 focus group discussions.

Preceptors completed demographic questionnaires at the beginning of each focus group discussion. To establish credibility of findings, two focus group discussions were held, one in April 2016 in Malawi and one in June 2016 in Lesotho. The focus group discussions were conducted in English as all eligible staff were fluent English speakers. A semi-structured discussion guide was created based on review of the existing literature on benefits and burdens of STLs, using an iterative process until consensus was achieved among the authors. Authors CAR and EMK, who each worked for a year as physicians at the study sites in parallel roles to focus group participants, were trained in focus group methodology and later moderated the focus group discussions in Malawi and Lesotho, respectively. A co-facilitator was present at both sessions to assist with recording the discussions and to ask clarifying questions. Each session lasted between 60 and 90 minutes. To establish confirmability of the findings, transcripts were audio-recorded, manually transcribed, de-identified, and double checked for accuracy. Facilitators de-identified data during transcription.

Transcripts were uploaded into Dedoose version 7.5.7 (Los Angeles, California), a qualitative analysis software [14]. To establish dependability of the findings, two authors experienced in qualitative research and who did not participate in the facilitation of the focus group discussions (HL and PS) independently coded the transcripts using an inductive approach. Thematic analysis was conducted to explore salient topics and recurrent themes that emerged during the focus group discussions. Qualitative data from focus group discussion transcripts were analyzed using a conventional content analysis approach. Collectively, the authors discussed overarching categories, themes, and representative quotations. This study was approved by the Baylor College of Medicine Children’s Foundation – Lesotho Institutional Review Board, the Malawi National Health Sciences Research Committee, and the Institutional Review Board at Baylor College of Medicine.

## Results

Of 18 eligible preceptors from low- and middle-income countries who had spent an average of 6–10 years in clinical practice, 10 participated in 2 focus group discussions (Table 1). In Lesotho 5 of 11 eligible preceptors (45.5%) and in Malawi 5 of 7 eligible preceptors (71.4%) participated in the focus group discussions.

**Table 1:** Demographics of international host preceptors in Malawi and Lesotho who participated in focus group discussions evaluating benefits and burdens of hosting short-term learners (n = 10).

Demographic		n (%)

Gender	Female	7 (70)
	Male	3 (30)
Nationality	Malawian	5 (50)
	Mosotho (Lesotho)	3 (30)
	Myanmar	1 (10)
	Nigerian	1 (10)
Country of Medical Training	Malawi	6 (54.5)
	Myanmar	1 (9.1)
	Nigeria	1 (9.1)
	South Africa	3 (27.3)
Years in Clinical Practice	2–5	1 (10)
	6–10	6 (60)
	>10	3 (30)
Clinical Role	Clinical Officer^a^	5 (50)
	Medical Officer^b^	4 (40)
	Pediatrician	1 (10)
Hours Per Month Spent Supervising Short-term Learners	0–20	3 (30)
	21–40	6 (60)
	41–60	1 (10)
Number of STLs Supervised in Past 12 Months	<5	2 (20)
	5–10	8 (80)
	11–15	0

^a^ Clinical officers are clinicians who had three years of medical education in Malawi after the high school equivalent and two years after the high school equivalent in Lesotho. ^b^ Medical officers are general practitioner physicians who have completed medical school and internship but no residency or sub-specialty training.

After an iterative analysis of the transcripts, several common themes emerged. These included perceived benefits to international host preceptors, perceived burdens to international host preceptors, perceived benefits to STLs, perceived burdens to STLs, ambivalence regarding balance of benefits between preceptors and STLs, and potential methods to enhance benefits to the international hosts.

## Perceived benefits to international host preceptors

### Professional positive impact

Preceptors cited several ways in which STLs benefited preceptors, including increasing their knowledge about disease processes, interpretation of diagnostic tests, and management, as well as new resources for learning. One participant remarked:

“With the conditions that are rare on this side but our colleagues who are visiting have knowledge and experience—we…benefit from the knowledge that they have…even the interpretation of diagnostics.”

Preceptors identified two specific ways that STLs changed usual day-to-day practice through the exercise of widened differential diagnoses and examination of patients. Several preceptors described how working with STLs encouraged them to widen the differential diagnosis when evaluating patients:

“They pick up on certain conditions, even the rare ones. The patient may present and you may be thinking of something different…[and the STL says] ‘but this looks like this and this instead,’ and you come up with a diagnosis together.”

Another described that the process of widening the differential and jointly coming up with a diagnosis is “beneficial to the patient.”

Two preceptors discussed how STLs’ practice of examining every patient “even when they [the patient] say they are fine” or when “they may be running around the exam room” was a change from their usual practice.

Learners also participate in continuing medical education as both attendees and lecturers while they are rotating at the clinics, which preceptors cited as a benefit to the clinic and patients.

### Personal positive impact

Many preceptors also expressed that they derive personal satisfaction from teaching STLs as they view teaching as central to the practice of medicine and fulfilment of their job duties:

“I enjoy teaching. I think it’s part of what is expected of us—part of our job description.”

“You teach and that is what medicine is about.”

One preceptor described the joy of working with a STL who saw a particular condition for the first time:

“When they see malaria for the first time, it’s like—wow!—you know? And, gosh, we see malaria all the time!”

## Perceived burdens to international host preceptors

### Negative impact on clinical flow

Preceptors also identified an inefficiency burden that learners place on preceptors. They perceived that supervision of STLs decreases efficiency due to having to explain what they were doing, correct STLs’ care, and especially when they have to serve as interpreters. One preceptor succinctly stated, “We give them our time.” One preceptor said:

“The speed that I see patients is totally different when I have a learner. I can be slow because I am explaining, translating, or interpreting back and forth. I am slower than I am when seeing patients alone.”

One preceptor agreed that working with STLs slowed down clinic flow but cautioned against rushing them:

“I wouldn’t say let’s rush the STLs to see patients…because ultimately we are going to push them to see patients on their own and at the end of the day, the patients are going to become mismanaged.”

### Negative impact on decision-making processes

Preceptors identified the burden of having to intervene in instances that could lead to patient harm, including when an STL may give incorrect medical advice or apply clinical guidelines inappropriately. For instance, one preceptor described a situation in which an STL incorrectly advised a patient’s mother to stop breastfeeding, in opposition to local guidelines; the preceptor unfortunately did not discover this incorrect advice until the patient came back for a follow-up appointment, and by that time the STL had completed their elective so the preceptor was not able to provide feedback to the STL.

Some also perceived that STLs undervalued and questioned preceptors’ clinical decision-making in a resource-limited context. For instance, one preceptor perceived a lack of respect from an STL:

“Most of the time, in Malawi, how we manage our patients depends on resources. And all the learners know is that if a patient has this, then what must be done is A, B, C, D, E. Where here maybe all we can do is A and B. Questioning is fine but sometimes it comes out as if ‘You don’t know what you are talking about.’”

## Perceived benefits to short-term learners

Preceptors recognized many benefits that STLs accrue, particularly through exposure to different populations, pathogens, and pathologies. As one preceptor summed up:

“They are exposed to different things they are not used to…so now if they see an African in the United States, they will have more ideas of what [disease] they could have.”

Preceptors also pointed out in regards to caring for children and adolescents with chronic illnesses, STLs see patients with more advanced disease and more complications than they might see in their home settings. Beyond exposure to different pathologies, many preceptors also voiced that STLs benefit from exposure to clinical decision-making in a resource-limited context:

“We are restricted in terms of resources, but we still manage to take care of our patients, and the STLs are surprised to see that.”

“The experience is totally different. In the Western world, [hospitals and clinics] are not as crowded as they are here. And there is a limited range of drugs that we use here. Even the way we do our minor procedures, it’s different. They get a lot [from seeing this].”

## Perceived burdens to short-term learners

### Financial burdens

Preceptors acknowledged that STLs may also experience burdens as they complete STGHEs. They recognized that STLs have to leave a familiar environment and, at times, take on the financial burden of travel:

“Coming here is a lot of sacrifice. They are leaving their family, their colleagues, and their homes. They come to a new place that they don’t know.”

“It’s big money…Not all of them who come are sponsored by their hospitals; some have to cover the cost of coming here.”

### Health burdens

Preceptors also mentioned personal health risks, such as exposure to malaria and tuberculosis, as well as security risks that STLs take during STGHEs:

“They are not sure of the behavior of the people here. They may not be sure of who is okay and who is not when they meet people in town.”

## Mutually beneficial learning

### STL-related factors

Preceptors felt that it was important that both sides benefit from STGHEs. Participants emphasized the need for both sides to learn from each other:

“They are trying to learn some things but also there are some things we might not be doing here that we want to learn from them. It should be a two-way thing. They benefit and we benefit.”

Preceptors agreed that both parties benefited from the experience, but they did not reach consensus whether learners or preceptors benefit more. Most said that it is hard to determine, but some recognized that individual STL factors, such as engagement and motivation, influence the balance of benefits. As one preceptor described:

“The ones who are serious enough with what they are here for, it will be a 50:50 kind of gain. But if they look like they are here casually, they are the ones that benefit more than we do from them.”

“We benefit more when they are eager to learn, to see this reality.”

Several mentioned that an STL’s level of training also correlated with the balance of benefits:

“For those who are already training towards the end of their studies, I think they are the best people to send and teach.”

### Institutional factors

Other preceptors commented on sending-institution factors, such as STL preparation, learning objectives, and the length of the STGHE, as influencing the balance of benefits. As one preceptor stated:

“For someone to come for a short time, say a couple of weeks, I think there will be a lot of mistakes. Two-week rotations should not be considered at all; it’s too short.”

### Relevance to home setting

In addition, some preceptors qualified their assessment on the balance of benefits with uncertainty of the relevance of the STGHE to STLs’ home settings. For instance, one preceptor remarked:

“I don’t know if it still benefits them or not because they go back to their usual setup, which has lots of resources, different medications, and their guidelines are different.”

One preceptor questioned whether the amount of effort preceptors invest in the STL experience is ultimately worthwhile:

“We have our own guidelines, we have our own way of doing things, and it’s totally different from the West. So, it’s like we are putting so much out. We are giving so much, which is okay…but I’m not sure how beneficial it is when they go back.”

## Suggested enhancements

### Pre-rotation preparation

International host preceptors suggested multiple ways to enhance benefits of STGHEs, including pre-departure preparation for STLs, improved orientation for preceptors, and specific learning objectives:

“We all need to understand what their objectives are. There are those that are coming with objectives and it’s not just ‘I’m going to Malawi for a month to save Malawi.’”

“I think it’s very important for the clinicians here to be informed about the program and expectations.”

### Lengthen duration of STGHEs

Preceptors also suggested making the duration of STGHEs longer:

“There’s a big difference between two weeks and four weeks. Not just for us, I think longer [duration of STGHEs] will be better for learners for their benefits to be greater.”

### Leverage cultural exchange for its learning potential

Preceptors also cite hosting STLs as an opportunity to learn more about other cultures:

“If I am going to host you, I need to know better your practices, your beliefs. Then I will be a better host. Otherwise I am totally blank on you.”

### Make expectations clear to learners

Preceptors perceived that mutuality of benefits is more likely to occur when STLs are interested and engaged learners and when expectations of mutuality of benefits are made clear to them. Preceptors commented that sending institutions should encourage STLs to teach at the clinics in order to leave something behind:

“It would be good to make it very clear to the visitors that the expected benefit is for both sides. We will count on you as part of the team.”

### Develop bidirectional exchange programs

To make the benefits more mutual, preceptors expressed the desire for an exchange program for learners from Malawi and Lesotho to visit the STLs’ countries of origin:

“I think there should be the program over there too. That way people also can go learn and come back to use the knowledge, [like] an exchange program.”

## Discussion

Ensuring that STGHEs are beneficial for both STLs and host sites is essential [15,16]. Preceptors in this study discussed the benefits and burdens of hosting STLs, the benefits and burdens to learners in doing a STGHE, and ways to improve benefits to the preceptors and ensure mutuality of benefits for both partners in STGHEs. Although a growing body of global health education literature calls for such reciprocity of benefits between hosts and learners [17,18], this is one of few studies to assess the host perspective on reciprocity of benefits and strategies to optimize it.

Preceptors identified ways in which STLs benefit preceptors, including increased knowledge and resources for learning from STLs, broadened differential diagnosis, and the satisfaction of teaching. This was similarly reflected in a study conducted in Germany by May et al. that showed preceptors enjoy teaching and bidirectional learning as it allows them to keep their medical knowledge up to date [19]. The preceptors in this study expressed similar sentiments, thus extending this finding to global health settings. Previous studies have also shown that host sites appreciate the service that STLs provide their patients and the sites themselves [19,20]. In addition, STGHE sites are thought to benefit from hosting STLs due to the perceived strengthening of their reputations in the international community as leaders in global health [16,21], which may lead to increased training opportunities for local staff, financial compensation, and opportunities for scholarship. Further, preceptors in this study noted that STLs with more advanced training tend to be more valuable to the host sites in terms of teaching and patient care, which sending institutions should note when selecting STLs for STGHEs.

Burdens to preceptors included perceived decreased clinical efficiency, having to intervene in instances that could lead to patient harm, and the perception that some STLs under-valued preceptors’ clinical decision-making. The fact that learners decreased preceptor efficiency has been noted in other studies [20]. Concerns about patient safety have also been raised, and the literature warns that some STLs perform tasks beyond their abilities or perform patient care without adequate supervision while abroad [19,22]. Finally, the perception that some STLs under-valued preceptors’ experience, particularly their ability to make complex clinical decision-making within resource-constrained settings, aligns with findings from another study in which interviewed preceptors commented on a general lack of respect for the preceptor [23]. Other studies have shown that hosting STLs may place a strain on teaching resources or compete with local learners [24].

Many benefits of STGHEs for STLs were recognized by the preceptors in our study, including exposure to different populations, pathogens, and pathologies; caring for patients with more advanced chronic disease; and exposure to clinical decision-making in a resource-limited context. This is supported by studies that have shown STLs gain enhanced knowledge of tropical diseases and decreased reliance on laboratory testing and imaging during STGHEs [2,3,4,5,6,7,8]. Further, studies have demonstrated that STLs that complete STGHEs during their medical training are more likely to work with underserved populations in their future careers [4,5,6]. The preceptors in our study did not mention this particular benefit, and when asked about the balance of benefits and burdens, they questioned the relevance of the clinical experience of the STGHE to STLs’ home settings. Thus, the preceptors may not receive feedback on the entirety of benefits that STLs experience, such as being more likely to work with underserved populations in their future careers.

Preceptors in this study also cited sacrifices that STLs make by being away from family, making financial commitments, and risks to their health and personal safety. This is supported by a number of studies that have suggested potential risks to students’ health and costs accrued by students are significant burdens [20,24,25,26]. Another study commented on STLs experiencing culture shock in a new environment, as well as being underprepared for ethically challenging situations in which they may be pressured to perform procedures with inadequate supervision [21,27]. Sending institutions and STLs should be aware of both the perceived and actual risk to STLs’ personal safety and take steps necessary to minimize these risks.

Factors that may improve benefits to preceptors were suggested, such as pre-departure preparation and longer duration of STGHEs. This finding adds to a growing body of literature on the importance of pre-departure preparation. Others have called for pre-departure preparation that includes both logistical preparation and cultural sensitivity training [28,29]. Careful consideration of international host preceptors’ input on pre-departure training may lead to more meaningful and impactful STGHEs for both the STL and the hosting institution. In addition, preceptors called for improved preceptor orientation and overall increased involvement in the electives to enhance the mutuality of benefits. Studies emphasize that equal partnership in designing electives, even in details like selecting the STLs and developing goals for the STGHEs, can help ensure that the program is set up with mutual respect and responsibility [18]. Thus, sending institutions should consider involving preceptors early in the development of the STGHE.

Preceptors in this study also proposed an exchange program, in which participants from both institutions could visit the other site in a bidirectional partnership. Others have suggested establishing such formal partnerships between sending and host institutions as one method to improve mutuality of benefits between institutions [19,30,31,32]. However, these reciprocal partnerships have proven difficult due to imbalanced power structures, limited opportunities for foreign trainees in the United States, and credentialing processes that make it difficult for U.S. institutions to allow foreign trainees to be involved in patient care [33]. Some programs have overcome these barriers by creating opportunities that involve shadowing, simulation medicine, and educational sessions for visiting learners from international sites [34,35,36]. Beyond bidirectional exchange of learners, other mechanisms for reciprocity have also been proposed to improve benefits to international host preceptors, such as a focus on capacity-building, requiring learners to bring skills or educational materials, and higher consideration of host priorities [9,16].

## Limitations

This study had a relatively small sample size; however, there were strict inclusion and exclusion criteria leading to limited eligible participants. The participants were representative of the larger pool of preceptors at the Baylor International Pediatric AIDS Initiative Clinical Centers of Excellence, two outpatient pediatric HIV clinics in urban settings. Further, there was a lower response rate in Lesotho than in Malawi, and less than 50% of eligible staff participated. This was due to the fact that in Lesotho staff work at many different outreach sites, so on any given day only about half of eligible staff are working at the main clinic. In addition, this study was conducted at pediatric clinical sites with a focus on HIV-related care, which may limit their generalizability to other disciplines. However, as the discussions did not involve HIV, these findings are likely applicable to other STGHE sites. Another limitation of this study is researcher biases and predispositions, as is always a limitation with qualitative research. Researcher bias may have been amplified in our study as the focus group facilitators were from high-income countries. However, their long-standing working relationship with the focus group participants may have mitigated the inherent bias of having foreign focus group facilitators. Finally, this study did not distinguish between medical student, resident, and fellow learners during the focus group discussions.

## Conclusion

International host preceptors in this study identified ways in which STLs benefit preceptors, including increasing knowledge and resources from STLs, broadened differential diagnosis, and the satisfaction of teaching. Preceptors perceived burdens including decreased efficiency, the need to intervene in instances that could lead to patient harm, and the perception that some STLs under-valued preceptors’ clinical decision-making in resource-limited contexts. They suggested factors that may improve benefits to preceptors, such as pre-departure preparation, longer duration of STGHEs, sending STLs who are further in their training, opportunities for bidirectional exchange, and improved preceptor orientation and involvement to ultimately enhance the mutuality of benefits between STLs and preceptors. These findings emphasize the need for sending institutions to prioritize mutuality of benefits between STLs and host sites when developing STGHEs. The principle of equity is central to the definition of global health [37]; as such, institutions that partner with or send STLs to international institutions should aim to make STGHEs equitable and mutually beneficial between STLs and host sites.
